# Finding gene regulatory network candidates using the gene expression knowledge base

**DOI:** 10.1186/s12859-014-0386-y

**Published:** 2014-12-10

**Authors:** Aravind Venkatesan, Sushil Tripathi, Alejandro Sanz de Galdeano, Ward Blondé, Astrid Lægreid, Vladimir Mironov, Martin Kuiper

**Affiliations:** Department of Biology, Norwegian University of Science and Technology (NTNU), N-7491, Trondheim, Norway; Department of Cancer Research and Molecular Medicine, Norwegian University of Science and Technology (NTNU), N-7489, Trondheim, Norway; Escuela Nacional de Sanidad, Instituto de Salud Carlos III, 28029 Madrid, Spain

**Keywords:** Knowledge management, Knowledge representation, Semantic Systems Biology, Semantic Web, RDF, SPARQL, Network extension, Gene expression, Transcription regulation, Protein-protein interaction, Transcription factor, Target gene interaction, Hypothesis assessment, Gastrin biology

## Abstract

**Background:**

Network-based approaches for the analysis of large-scale genomics data have become well established. Biological networks provide a knowledge scaffold against which the patterns and dynamics of ‘*omics*’ data can be interpreted. The background information required for the construction of such networks is often dispersed across a multitude of knowledge bases in a variety of formats. The seamless integration of this information is one of the main challenges in bioinformatics. The Semantic Web offers powerful technologies for the assembly of integrated knowledge bases that are computationally comprehensible, thereby providing a potentially powerful resource for constructing biological networks and network-based analysis.

**Results:**

We have developed the Gene eXpression Knowledge Base (GeXKB), a semantic web technology based resource that contains integrated knowledge about gene expression regulation. To affirm the utility of GeXKB we demonstrate how this resource can be exploited for the identification of candidate regulatory network proteins. We present four use cases that were designed from a biological perspective in order to find candidate members relevant for the gastrin hormone signaling network model. We show how a combination of specific query definitions and additional selection criteria derived from gene expression data and prior knowledge concerning candidate proteins can be used to retrieve a set of proteins that constitute valid candidates for regulatory network extensions.

**Conclusions:**

Semantic web technologies provide the means for processing and integrating various heterogeneous information sources. The GeXKB offers biologists such an integrated knowledge resource, allowing them to address complex biological questions pertaining to gene expression. This work illustrates how GeXKB can be used in combination with gene expression results and literature information to identify new potential candidates that may be considered for extending a gene regulatory network.

**Electronic supplementary material:**

The online version of this article (doi:10.1186/s12859-014-0386-y) contains supplementary material, which is available to authorized users.

## Background

Cellular signaling cascades support the transmission of information from external signals (e.g. hormones) to distinct cellular responses, for instance changes in gene expression. Gene expression is controlled by a network of highly interconnected proteins known as transcription regulators [1,2]. There is a large array of transcription regulators including general transcription factors, sequence-specific DNA binding transcription factors (DbTFs), various transcription co-factors and chromatin modifiers [3,4]. Research in the field of gene expression is particularly important because various aberrations of this process have been implicated in the development of diseases, including cancer. Consequently, the research in this field has now generated a huge volume of information, which is certain to grow in the years to come. However, this information and the associated data are scattered across a multitude of resources in a variety of formats, which makes it a challenge to obtain a comprehensive access to all information necessary to answer questions that biologists working in this field may pose.

In general, the formulation and assessment of biological hypotheses against prior knowledge fundamentally relies on efficient knowledge integration that interlinks information and knowledge at various levels in standardized formats, after which the best-supported hypotheses can be selected for testing in wet-lab experiments. Therefore, the development of technologies for knowledge integration and representation has evolved into a major research area [5,6].

In recent years the Semantic Web has emerged as one of the most promising solutions to high scale integration of distributed resources. The Semantic Web initiative [7] essentially aims at transforming the current Web into a global reasoning and semantics-driven knowledge base. The Semantic Web is founded on a stack of technologies such as the Resource Description Framework (RDF) [8], RDF Schema (RDFS) [9], Web Ontology Language (OWL) [10] and the SPARQL Query Language (SPARQL) [11]. RDF, part of the basis of the stack, models data as a directed graph composed of so-called triples, each comprising two nodes (the subject and the object) connected by an edge (the predicate). All these technologies use the Uniform Resource Identifiers (URI) to identify real-world objects and concepts and the Hypertext Transfer Protocol (HTTP) for communication. The SPARQL querying language allows for the retrieval of triples of interest (a sub-graph) from an arbitrary set of RDF graphs that may reside at various locations on the Internet.

Ontologies, though introduced to the field of knowledge management long before the advent of the Semantic Web, have become an indispensable tool for practical implementations of semantic web technologies by providing a common understanding for people and computers alike, and may be regarded as part of the toolbox of the Semantic Web. In the field of biomedical research, the Open Biomedical Ontologies (OBO) Foundry [12] provides a set of guidelines to structure the coordinated development of bio-ontologies. Bio-ontologies developed following the guidelines of the OBO Foundry are becoming widely used by the life science community. The Gene Ontology (GO), a prominent example of this [13], provides a unified representation of properties of genes and their products. Furthermore, the Gene Ontology Annotation (GOA) project [14] facilitates unambiguous annotation of gene products with GO terms covering molecular function, cellular component and biological process aspects.

We are currently witnessing a growing use of semantic web technologies for the management of biological concepts and for providing a scaffold for integrating concepts and data from disparate biological databases [15-17]. In this vein we have developed the Gene Expression Knowledge Base (GeXKB), to serve the needs of researchers working in the field of gene regulation. We were motivated by the following considerations:

1. Even though SPARQL supports federated querying, this mode presents an additional hurdle for a biologist.
2. Querying distributed and typically very large resources takes long execution times.
3. The currently available reasoners are still too sluggish to be deployed on very large graphs, in particular when rule chaining is involved.
4. The resources necessary for adequately answering specific questions are not always found in the available triple stores.

GeXKB accommodates the field of gene expression regulation by seamlessly integrating the most relevant ontologies and databases, using semantic web technologies (preliminary results appeared in a conference paper [18]). GeXKB was developed in close collaboration with end users who provided requirements and use cases. The use cases were taken from the domain of gastrin hormone response pathways, in particular gastrin-mediated gene regulation, introduced below.

### Use cases

Several biological questions were formulated in the context of the gastrin response pathways. Gastrin is a gastrointestinal peptide hormone, which, similar to many other extracellular signals such as e.g. growth factors, plays a crucial role in both normal and pathological processes. After binding to the Cholecystokinin 2 receptor (CCK2R), gastrin triggers the activation of multiple intracellular signaling pathways and transcription regulation networks culminating in the regulation of numerous genes. We previously performed an extensive genome-wide gene expression time-series experiment on gastrin-treated rat AR42J cells [19] (the ArrayExpress database [20], accession number: GSE32869). This work allowed us to identify genome wide changes in mRNA levels in response to gastrin, serving as an experimental reference for our study. In addition, we used a map of gastrin responsive intracellular signaling and transcription regulation networks, which we built previously through an exhaustive search for experimental evidence reported in literature [21]. This map was taken as a point of departure to identify new proteins that should be considered as putative network extensions. We reasoned that, given the knowledge sources integrated into GeXKB, queries based on our biological questions should yield both well established and new gastrin response network participants. In total we developed 6 queries (identified as Q1 through Q6, see Query formulation section) for the following four use cases:

#### Use case I: Finding protein candidates involved in regulation of transcription factor CREB1

The cAMP response element binding protein 1 (CREB1) is a specific DNA binding transcription factor. It is known to be under the control exerted by multifarious regulator complexes that include DbTFs, co-factors and kinases. We were interested in retrieving an exhaustive overview of possible regulators of CREB1.

#### Use case II: Identifying repressors of NFκB1 and RELA that undergo proteasomal degradation

**Figure 1:**
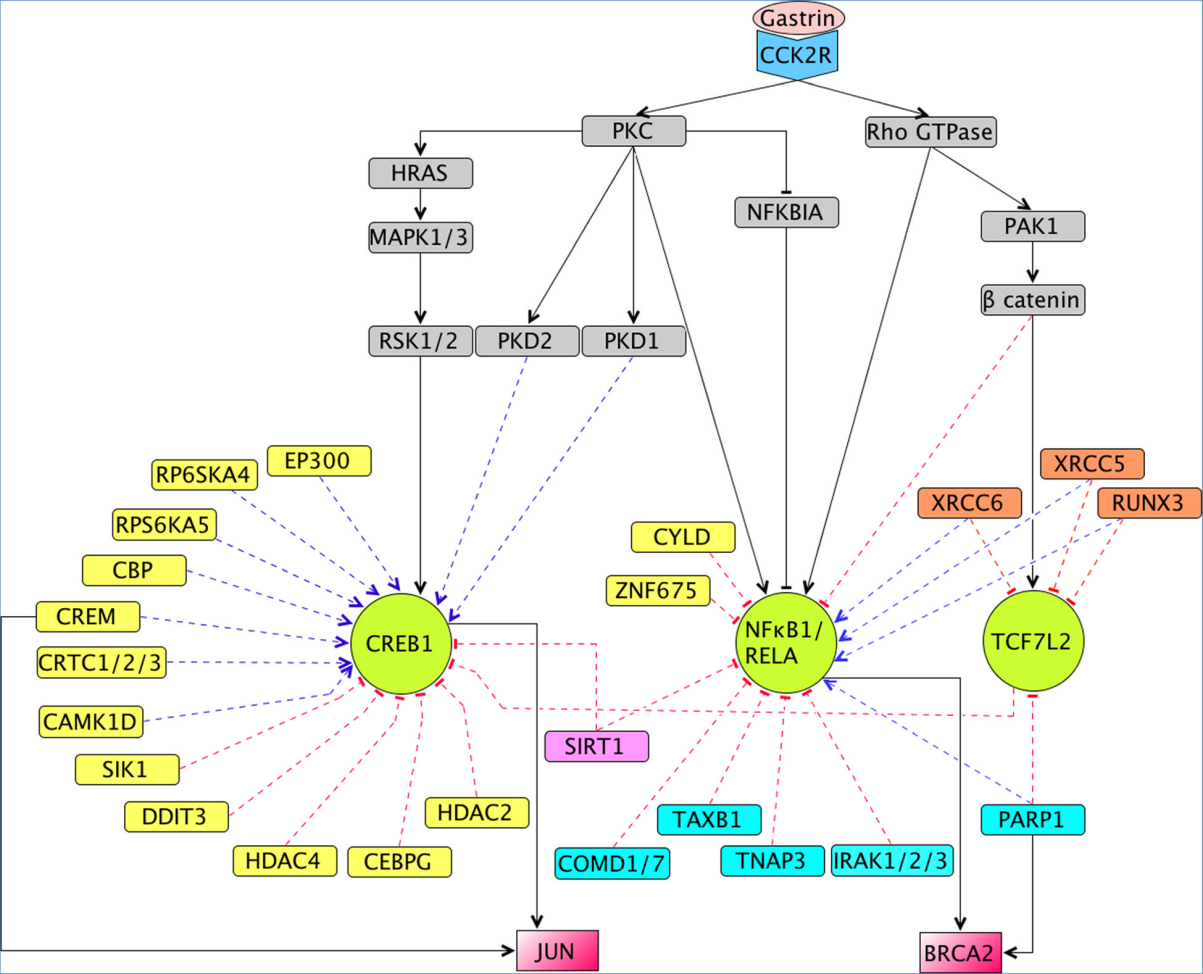
**Core CCK2R network and novel candidate regulators.** The core of the gastrin mediated signal transduction network (CCK2R), and the novel candidate regulators resulting from our queries are shown. The CCK2R DbTFs that were targeted in our queries are colored light green. The network components in grey and the solid lines connecting them are part of the core CCK2R network and documented as regulators of the CCK2R DbTFs and respond to gastrin. The dotted lines represent new relations identified by the queries which could be verified against literature: blue pointed arrows denote ‘activation or positive influence’ and red bar-headed arrows depict ‘repression or negative influence’. CREB1 candidate regulators identified through Q1, Q2 and Q3 are colored yellow. Candidate regulators of NFκB1 identified through Q4 are colored turquoise, and candidate regulators of TCF7L2 identified through Q5 are colored orange. The target genes shared by the CCK2R DbTFs (CREB1 and NFκB1) and the DbTF candidates identified through Q6 are colored light red (JUN and BRCA2) and their connections are shown as solid arrows.

NFκB1 and RELA are members of the NFκB transcription factor family known to be involved in regulating apoptosis, proliferation, and immune responses [22]. Gastrin dependent regulation of these transcription factors reportedly is mediated through PKC and Rho GTPase signaling cascades [23,24] (Figure 1). The activity of NFκB transcription factors is under the control of a family of inhibitors, known as ‘inhibitors of kB’ (IkB), which sequester NFκB in the cytoplasm and thereby keep these transcription factors in their inactive state [25]. Proteasomal degradation of IkB factors results in restoration of the active state of the NFκB and promotes its import to the nucleus. In order to gain detailed mechanistic insights in NFκB regulation, we were interested in retrieving proteins that contribute to NFκB down-regulation, and at the same time have functions related to proteasomal degradation.

#### Use case III: Listing components that function as repressors for TCF7L2 and activators for NFκB1 or CREB1

DbTFs are implicated in different cellular processes in the gastrin response signaling cascade. TCF7L2 plays a central role in gastrin mediated cellular migration [26], whereas NFκB1 and CREB1 are pivots of regulation of gastrin dependent immune responses and proliferation, respectively [27,28]. Proteins that function as repressors for one transcription factor and activators for another can be of potential significance for cellular decision making.

#### Use case IV: Identification of genes that are shared targets of DbTF regulators and the DbTFs described in use cases I-III

DbTFs are central to the regulation of gene transcription, which in turn plays a key role in determining gene expression levels. Often, several DbTFs act together in the regulation of transcription of a specific gene. To enhance our understanding of mechanisms involved in gastrin mediated cellular responses we were interested in retrieving shared target genes of CREB1, NFKB1, TCF7L2 and the regulators of these DbTFs.

## Methods

### GeXKB construction

GeXKB was conceived as an easily extensible knowledge base consisting of a core to which any number of optional resources could be easily added (See Results/GeXKB, for a detailed description of the contents).

**Figure 2:**
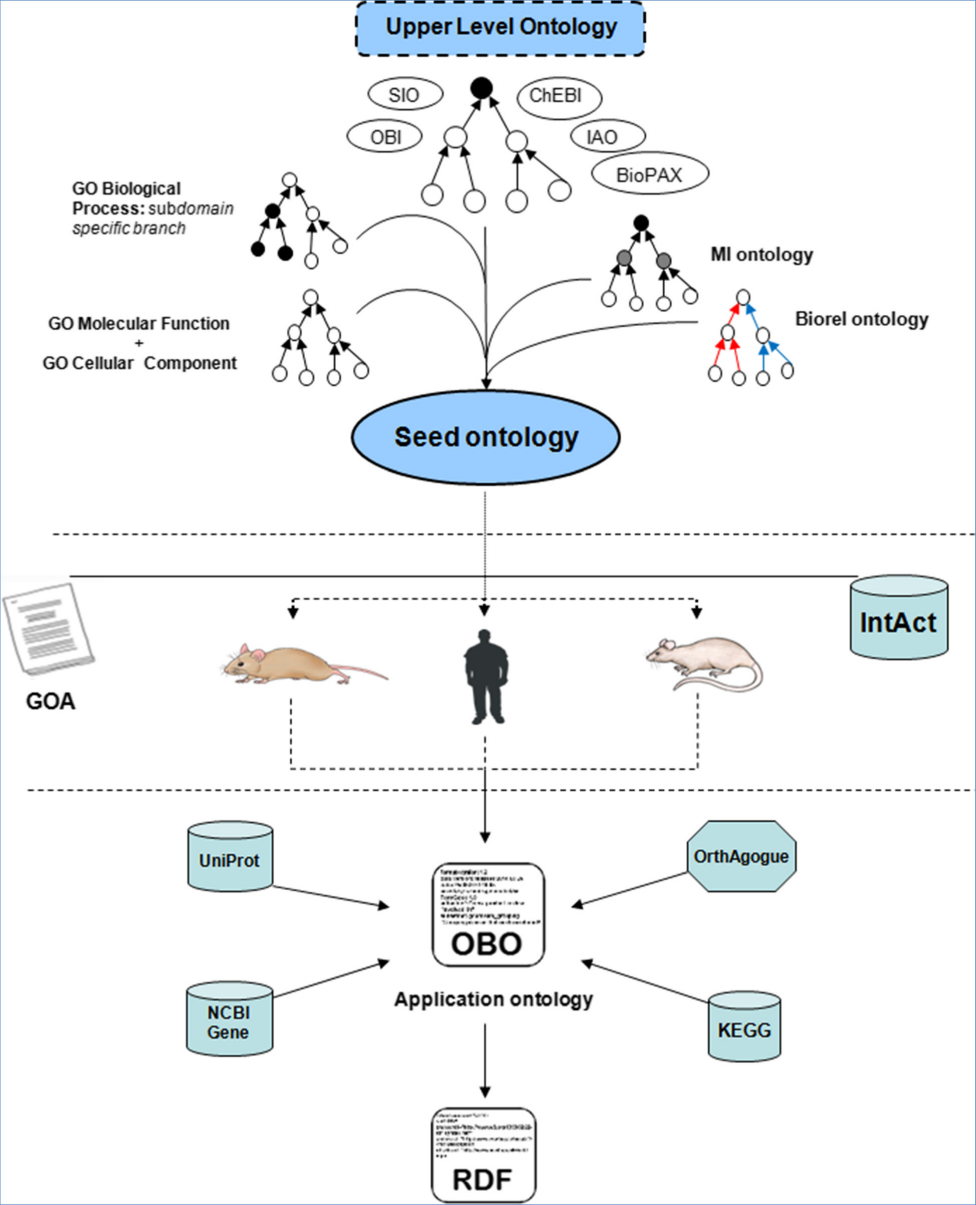
**The data integration pipeline.** The integration starts by generating an Upper Level Ontology, which is then linked with the different ontologies: GO (Biological Process, Molecular Function and Cellular Component fragments), the MI ontology and the Biorel ontology, forming a seed ontology. Mouse, human and rat-specific data are integrated from Gene Ontology Annotation files and IntAct. Next, these species-specific ontologies are merged and additional data is integrated including protein information (UniProt), pathway annotations (KEGG), basic information for genes (NCBI) and orthology relations for proteins (orthAgogue). The final ontology is available in OBO and RDF formats.

**Figure 3:**
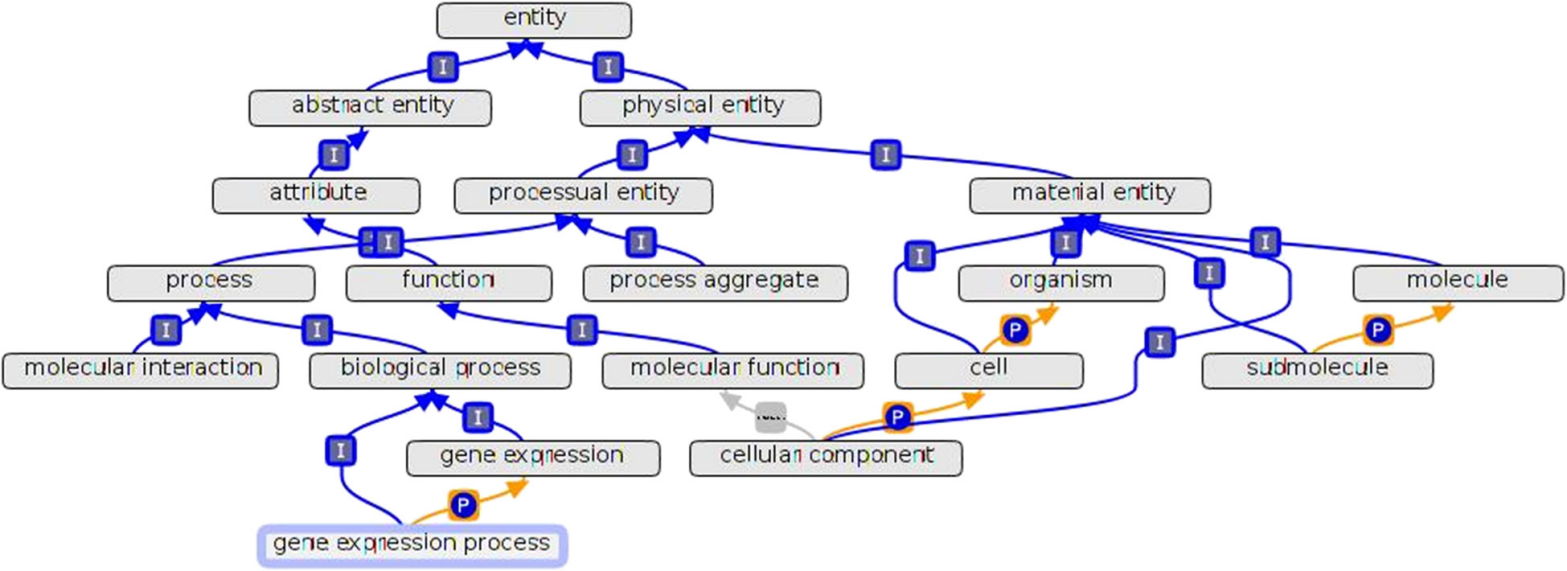
**Upper Level Ontology (ULO).** The ULO was developed on the basis of terms imported from other ontologies. The three application ontologies have structurally identical ULOs, differing only in the sub-domain specific terms. The figure illustrates the ULO structure of GeXO.

The construction involves 1) the development of three application ontologies that form the core of GeXKB, 2) conversion of optional resources to RDF, 3) uploading the ontologies and the optional resources to a triple store to make them accessible through a SPARQL endpoint, 4) inferring and adding to the store new triples supported by the explicitly asserted ones to increase the power and flexibility in querying. The 4 steps in detail:

Step 1: The GeXKB ontologies are generated by an automated data integration pipeline (Figure 2) that relies on the ability to programmatically manipulate ontologies with the ONTO-PERL API [29]. This pipeline allows the ontologies to be easily updated. First, a concise upper level ontology (ULO) is assembled from terms imported from other ontologies (Figure 3). Next, fragments of the GO ontology, a fragment of the MI ontology [30] and the Biorel [31] ontology are linked to the ULO. The result is three ontologies referred to as the seed ontologies. Further sets of proteins are retrieved from the Gene Ontology Annotation files by association with the Biological Process terms present in each of the seed ontologies. These sets of proteins (referred to as ‘core’ proteins) are used subsequently as a basis to select by association additional proteins from IntAct protein-protein interactions [32], KEGG pathways [33] and binary orthology relations as predicted by the orthAgogue utility [34], a high performance C++ implementation of OrthoMCL [35]. Finally, protein modifications, basic gene information and associations with Cellular Component and Molecular Function terms from GO are added from UniProtKB [36], NCBI Entrez [37] and the Gene Ontology Annotations, respectively (see Additional file 1 for the full set of term types in GeXKB). The pipeline finally outputs the three application ontologies in the OBO [38] and RDF [8] formats.

The mappings provided by UniProtKB [39] are used for inter-conversion of IDs and names in the core GeXKB. Entities which cannot be mapped in this way are omitted. All the identifiers in GeXKB ontologies are in the form *nameSpace*:*ID* in the OBO files and *nameSpace*_*ID* in the RDF files. Original IDs are used throughout if available. IDs for modified residues are constructed by replacing spaces with underscores in the corresponding names. Original name spaces are used for the imported ontological terms. The only ontological terms constructed specifically for this project are GeXO:0000001, ReXO:0000001 and ReTO:0000001. These three terms are modelled by analogy with the term ‘cell cycle process’ in GO. The name spaces used for other term types are as follows: ‘UniProtKB’ for protein terms, ‘KEGG’ for pathway terms, ‘NCBIGene’ for gene terms, ‘NCBITaxon’ for taxon terms, ‘SSB’ for modified residue terms and ‘intact’ for protein-protein interactions terms. Apart from the generic subsumption and partonomy, 10 more specific relation types are used to construct GeXKB ontologies (see Additional file 1).

Step 2: The optional resources are converted to RDF with the use of simple Perl scripts. Documented information about the functional interaction of DbTFs with their target genes is added from: a) the PAZAR database [40], an open source framework that serves as an umbrella to bring together datasets pertaining to transcription factors and regulatory sequence annotations; b) the Human Transcriptional Regulation Interactions (HTRI) database [41], an open-access database that serves as a repository for experimentally verified human transcription factor - target gene interactions; c) TFactS [42], a database that catalogs curated transcription factor - target gene interactions; and d) TFcheckpoint [43], a database that compiles curated information on human, rat and mouse DbTF candidates from many different database resources. As described above (step 1), entities from these resources are filtered based on the ID mapping file provided by UniprotKB. (Additional file 1 for the number of DbTFs and target genes per resource).

Step 3: All the RDF files are uploaded to an instance of the OpenLink Virtuoso data storage engine [44] as separate graphs using Virtuoso’s iSQL interface. The graphs are made accessible by SPARQL via a web page query form which offers a collection of pre-assembled queries to aid novice users [45].

Step 4: The inference process is performed by using the SPARQL update language (SPARUL) [46] as described in [31]. The graphs containing pre-computed inferences is suffixed with ‘-tc’ (e.g. ReTO-tc, where ‘tc’ stands for total closures).

### Query formulation

All biological questions for the use cases (see section: Use cases) were converted to SPARQL queries targeting the *Homo sapiens* information in GeXKB.

#### Use case I

**Figure 4:**
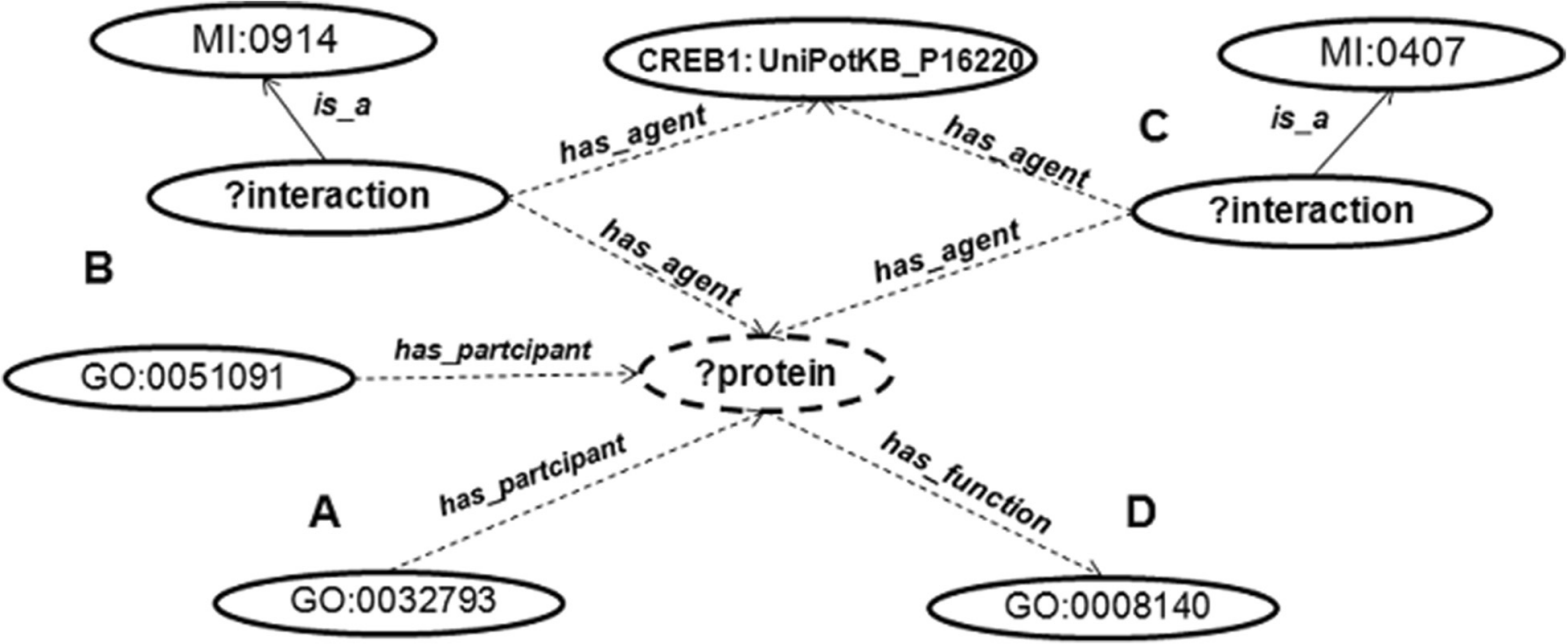
**Conceptual model of Q1.** The figure displays the different concepts, ontology terms and relationships that together form a graph that was used as a SPARQL query to find matching patterns in GeXKB. The query specifies proteins that **A)** exhibit positive regulation of CREB transcription factor activity (GO:0032793); **B)** exhibit positive regulation of sequence-specific DNA binding transcription factor activity (GO:0051091) and are linked to the CREB1 protein through an association (MI:0914); **C)** are linked to the CREB1 protein through a direct interaction (MI:0407); and **D)** have function cAMP response element binding protein binding (GO:0008140).

To address use case I, three queries were formulated (Q1-Q3, Additional file 2) that return positive and negative regulators and chromatin modifiers of CREB1 (UniProt accession: P16220, commonly referred to as “CREB”). Query Q1 retrieves proteins that are involved in the activation of CREB1. To achieve this, the query combined different terms that suggest the activation of CREB1. First of all, we used the ReTO and ReTO-tc graphs as default graphs for the queries as they are suitable to query nuclear transcriptional processes. Next, the GO terms *positive regulation of CREB transcription factor activity* (GO:0032793) and *cAMP response element binding protein binding* (GO:0008140) were included in the query. These terms suggest direct association with the process of regulating CREB1. Additionally, the term *direct interaction* (MI:0407) was included in the query to retrieve proteins that interact directly with CREB1. Then, to widen the breadth of the query, the broader GO term *positive regulation of sequence*-*specific DNA binding transcription factor activity* (GO:0051091) was included. However, in this case only proteins that have a *physical association* (MI:0914) with the CREB1 protein were considered, thus reducing the number of false positives (see Figure 4).

Similarly, Q2 retrieves proteins involved in the repression of CREB1 protein. For this query, proteins associated with biological process terms *negative regulation of CREB transcription factor activity* (GO:0032792) and *negative regulation of sequence*-*specific DNA binding transcription factor activity* (GO:0043433) were used.

The query Q3 specifies chromatin modifiers that are involved in the regulation of CREB1. It retrieves the union of proteins associated with the molecular function terms *histone acetyltransferase* (GO:0004402) and *histone deacetylase* (GO:0004407) activity that are involved in the biological process *regulation of sequence*-*specific DNA binding transcription factor activity* (GO:0051090), and are interacting with the CREB1 protein. Other than providing putative network components, these queries also serve to demonstrate the utility of targeting relations obtained through the inferencing process. By using the ReTO-tc graph, we were able to include implicit knowledge statements in the query output, meaning ontology term relationships not directly annotated to proteins, but linked to them through the inferencing process (see section: GeXKB construction).

#### Use case II

Use case II is represented by Q4, which was constructed similar to the previous queries by using a combination of terms. First, the GO term *negative regulation of NFκB transcription factor activity* (GO:0032088) was chosen as the central term, as this would retrieve all proteins annotated as negative regulators of NFκB1 and RELA. Next, GeXKB was explored to identify terms that suggested an involvement with proteasomal degradation. Several terms were identified: *ubiquitin ligase complex* (cellular component: GO:0000151), *ubiquitin binding* (molecular function: GO:0043130), *ubiquitination reaction* (interaction type: MI:0220), and *ubiquitin mediated proteolysis* (KEGG pathway: ko04120). The SPARQL *union* construct was used to formulate a combination of the central term and the additional set of terms.

#### Use case III

Query Q5 represents use case III, but for this query no terms specifically suggesting negative regulation of TCF7L2 were found (contrary, for instance, to Q4 where a specific GO term was used to retrieve negative regulators of NFκB protein). Hence, Q5 was formulated by using generic terms that indicated a dual role of proteins. Consequentially, Q5 retrieves proteins that interact with the TCF7L2 protein (UniProt accession: Q9NQB0) and are further annotated with the terms *negative regulation of sequence*-*specific DNA binding transcription factor activity* (GO:0043433), and *positive regulation of sequence*-*specific DNA binding transcription factor activity* (GO:0051091).

#### Use case IV

Use case IV was investigated by first identifying DbTFs among the results obtained for queries Q1, Q2, Q4 and Q5. This was done by extending these queries and using the TFcheckpoint graph for DbTF identification.

Next, Q6 was formulated to retrieve from the TFactS, PAZAR and HTRIdb graphs target genes shared between the query DbTFs (CREB1, NFKB1 and TCF7L2) and the DbTFs identified above.

## Results

### GeXKB

GeXKB utilizes the knowledge representation features offered by RDF and builds on previous efforts to use semantic web technologies for the integration of knowledge [47-51]. GeXKB supports the three model organisms *Homo sapiens*, *Mus musculus* and *Rattus norvegicus*. Currently GeXKB is composed of three application ontologies integrating only primary resources which are regularly updated; four secondary resources containing DbTF-target gene relations (not necessarily up to date); and ID mappings to support querying.

The knowledge base is hosted by a triple store and can be queried with SPARQL.

**Figure 5:**
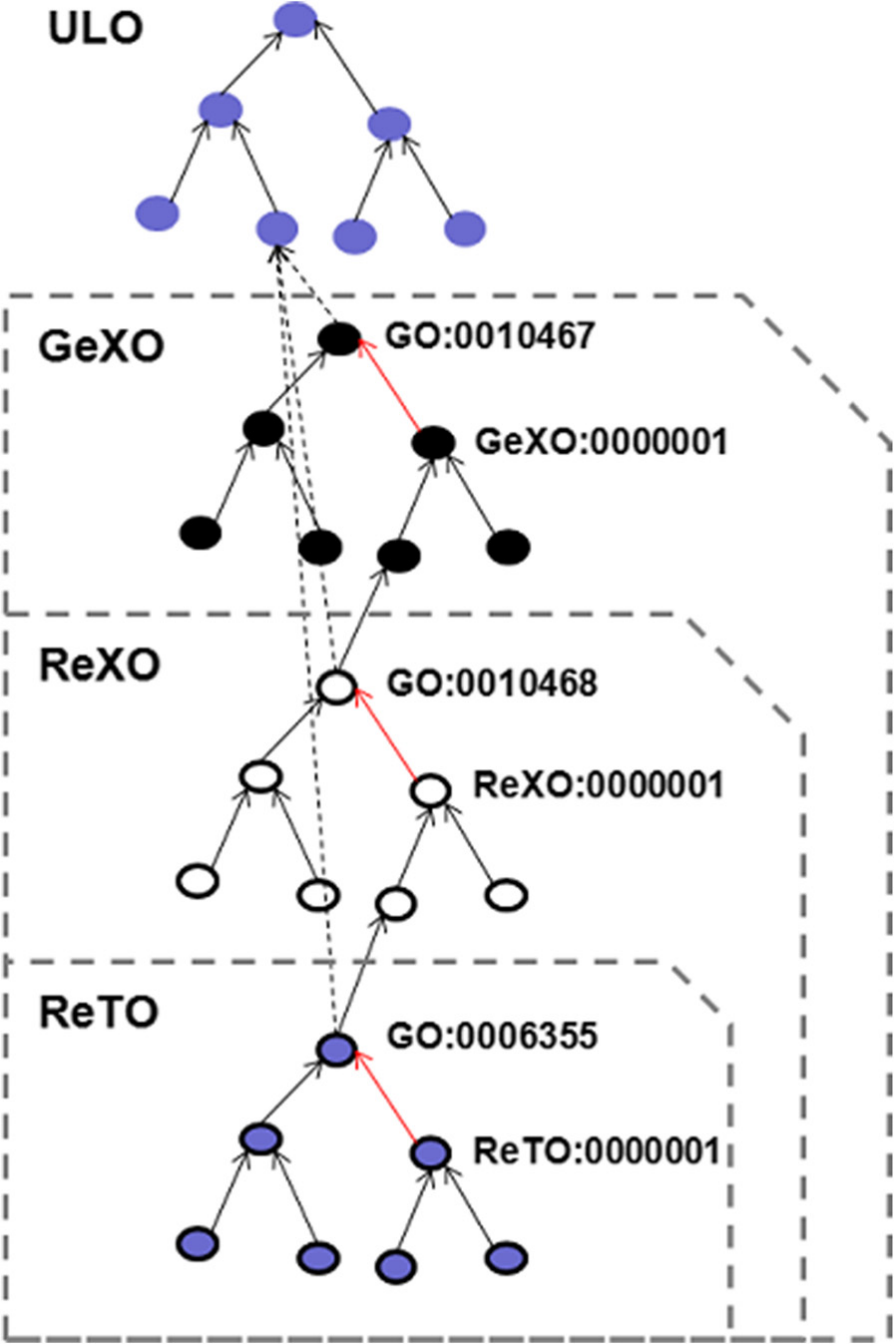
**GeXKB ontologies.** The illustration shows the layout of the nested GeXKB ontologies (GeXO, ReXO and ReTO).The blue nodes represent the upper level ontology (ULO), the common root of the three ontologies. The black and red edges depict ‘is_a’ and ‘part_of’ relations, respectively. The three ontologies cover an increasingly wide domain. Each GO sub-domain term (e.g. GO:0010467; denoting ‘gene expression’) and its descendants are linked to the ULO as a subclass of ‘Biological Process’ represented by the ‘dotted edges’.

To satisfy the requirements of end users, three nested application ontologies (see Figure 5) were developed: the Gene eXpression Ontology (GeXO, 89735 terms, 455859 relationships); the Regulation of Gene eXpression Ontology (ReXO, 77610 terms, 382721 relationships); and the Regulation of Transcription Ontology (ReTO, 70222 terms, 341963 relationships). All the three ontologies are 18 levels deep and ‘is_a’ complete. These application ontologies are knowledge bases in their own right since, unlike domain ontologies, they include not only ontological terms but experimental data as well (see below). This unique design allows for fast execution of even complex queries. The availability of three ontologies varying in breadth allows to easily define the specificity while querying.

The GeXKB ontologies share a common Upper Level Ontology (ULO), which is built ‘on the fly’. It is not available as an independent artifact in contrast with upper level ontologies like BFO, and it solely serves to ‘glue’ together the various components within an application ontology (Figure 3). The ULO was developed on the basis of SIO [52] (14 terms). A small number of additional terms (1 or 2 per ontology) from BioPAX [53], ChEBI [54], IAO [55], PSI-MOD [56], and OBI [57] are used to provide an interface between the SIO terms and the data, when needed. The ULO is merged with the GO through sub-domain-specific fragments of the Biological Process branch, and the complete Molecular Function and Cellular Component branches. More specifically, the GO terms ‘gene expression’ (GO:0010467), ‘regulation of gene expression’ (GO:0010468) and ‘regulation of transcription, DNA dependent’ (GO:0006355) with all their descendants were imported into GeXO, ReXO and ReTO, respectively. Additionally, the molecular interaction data is supported by the ‘interaction type’ branch of the Molecular Interaction (MI) ontology [30]. The Biorel ontology [31], an extension of the Relational Ontology [58], is included to provide additional vocabulary to logically link entities with relation attributes such as transitivity, reflexivity, subsumption, and priority over subsumption.

The GeXKB ontologies are protein-centric, and they are populated with proteins from GOA, IntAct, KEGG, and orthology relations by the filtering and aggregation procedure described in the Methods section. The essential information available about proteins includes GOA associations, IntAct protein-protein interactions, KEGG pathways, protein modifications, orthology relations and, when available, the corresponding genes (see Additional file 1 for the number of different term types). Gene terms are present in the ontologies only if UniProtKB provides a reference to NCBI Entrez, and consequently the number of gene terms in the ontologies is considerably lower compared to the number of protein terms (Additional file 1).

Although RDF is efficient in integrating data, it has limited expressivity and it was not conceived to perform inferencing tasks. In GeXKB this limitation is partially overcome by the use of a semi-automated reasoning approach developed in [31]. This approach allows the inference of new relationships on the basis of relationships explicitly asserted in GeXKB, based on five inference rules, namely reflexivity, transitivity, priority over the subsumption relation, superrelations and compositions [59]. The application of this procedure has resulted in approximately a 7 fold increase in the number of triples.

A major effort of the Semantic Web community aspires to make resources available as part of the Linked Data cloud [60]. We have taken initial steps towards making the GeXKB resource Linked Data-compatible, therefore we re-use original IDs for all entities in GeXKB and we use a common namespace (http://www.semantic-systems-biology.org) for all URIs. This solution combines the benefits of faster query execution and familiarity of the IDs for users. For instance, GeXKB can be queried using NCBI Gene IDs or UniProt accessions to retrieve information pertaining to a gene or protein of interest.

### Use cases

**Figure 6:**
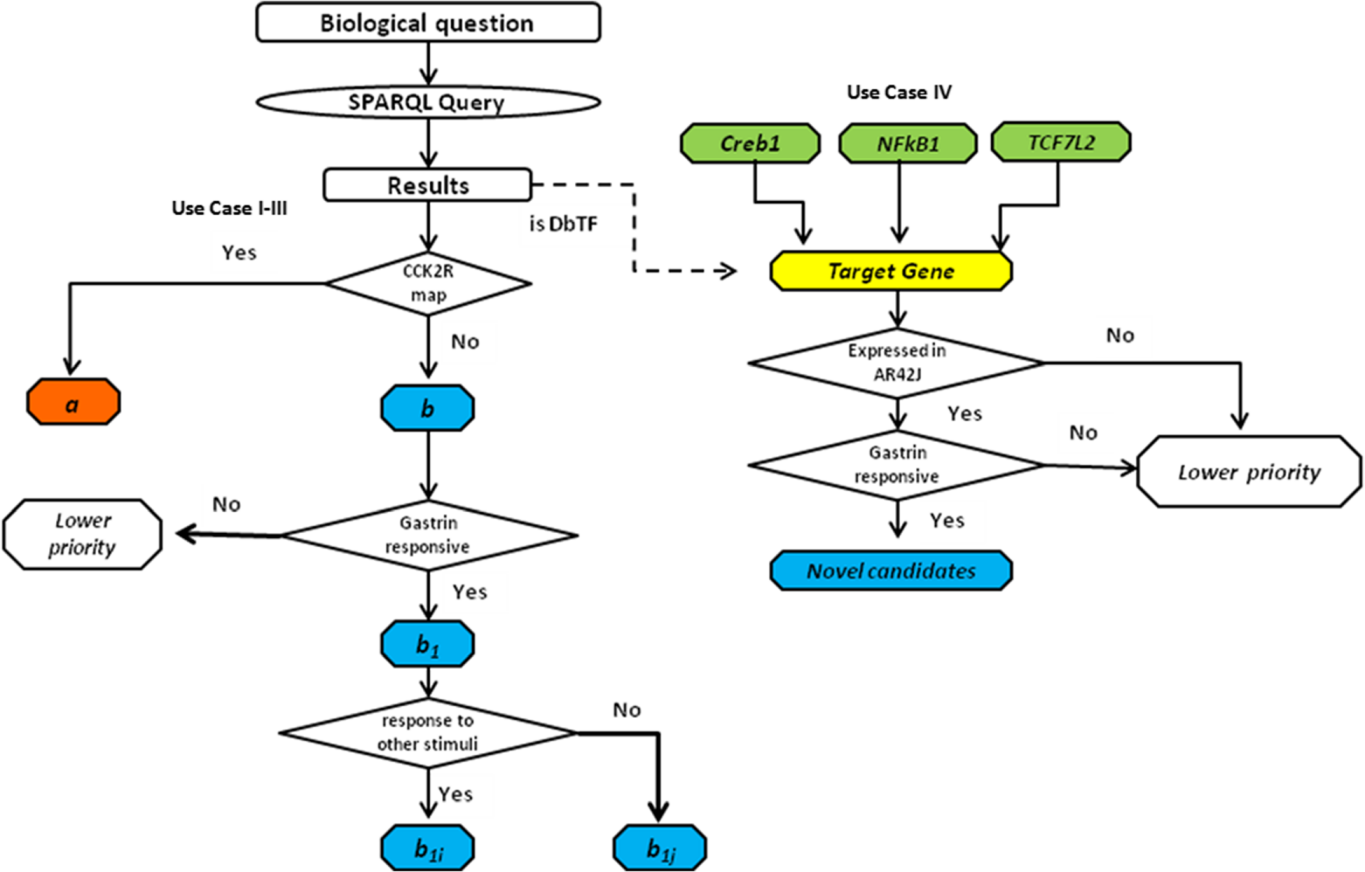
**Result evaluation.** The flowchart illustrates the evaluation of the results returned for the use cases I through IV. The proteins retrieved for use cases I, II and III were first classified based on their presence in the CCK2R map, constituting two groups a and b. The proteins under group b were further evaluated based on evidence of gastrin induced regulation constituting sub-group b_1_. Proteins in b_1_ were prioritized based on literature evidence implicating them to respond to stimuli other than gastrin (b_1i_ ), and proteins not reported to be responsive to other stimuli (b_1j_). Proteins qualifying both as b_1_ and b_1i_ were considered to be the most promising new putative network members. Similarly, the target genes returned for use case IV were evaluated for their expression in the AR42J cell system and whether these target genes were gastrin responsive. Genes that satisfied both criteria were prioritized as putative network members.

The results returned for uses cases I through III were investigated for their relevance to the gastrin response network [21] by categorizing them into two disjoint sets: a) proteins that have already been documented as members of the gastrin response network, and b) potential novel components of the gastrin response network. Within the latter a subset of regulators responsive to gastrin, referred to as *b*_*1*_ below, was identified on the basis of transcriptomic data from a 14h time series gastrin response data set [19]. Within *b*_*1*_ two disjoint subsets were defined – proteins known to be responsive to stimuli other than gastrin, and those not known, designated *b*_*1i*_ and *b*_*1j*_ respectively. The purpose of this classification was to prioritize the putative components. For instance, *b*_*1*i_ proteins were given higher priority as new putative members of the gastrin response network members due to the available evidence from literature, whereas proteins in category *b*_*1j*_ are still potentially interesting for future laboratory work, but with a lower priority. Finally, in use case IV the results returned for Q6 were assessed based on whether the genes regulated by the DbTFs in the query are expressed in the AR42J cell line and whether their expression changed in response to gastrin stimulation (see Figure 6). The six SPARQL queries and the results of use cases I - III are available in the Additional files 2 and 3 respectively.

All queries combined returned 148 putative regulators and 20 target genes. Queries Q1, Q3, Q4 and Q5 were launched against RDF graphs containing inferred triples (the tc graphs, see Methods). Q1 returned 37 proteins, 24 of them obtained by inferencing; Q4 returned 32 proteins with 17 proteins resulting from inferencing. In contrast, the results produced by Q3 and Q5 were solely based on the inferred triples, and yielded 21 and six proteins, respectively. Table 1 shows the breakdown of the number of proteins and genes returned for the six queries.

**Table 1:** **SPARQL query results**

	**Use case I**			**Use case II**	**Use case III**	**Use case IV**
	**Q1**	**Q2**	**Q3**	**Q4**	**Q5**	**Q6**
**Asserted components**	13	52	-	15	-	20
**Inferred components**	24	-	21	17	6	n/a
**Intersection**	3	0	0	0	0	n/a
**Total**	37	52	21	32	6	20

The table shows the breakdown of results returned from the six SPARQL queries that were part of use case I - IV. **Asserted components**: the number of proteins retrieved by direct statements; **Inferred components**: proteins retrieved by inferred statements; **Union**: the number of proteins retrieved by using a combination of asserted and inferred statements in the queries; **Intersection**: the number of proteins that are common between asserted and inferred statements; **Total**: the total number of proteins and genes retrieved by the six queries. Note: n/a – not applicable.

Considering the relevance categories described above, the 110 proteins identified in use case I include 52 proteins qualified as *b*_*1*_, 16 proteins as *b*_*1i*_ and 36 proteins as *b*_*1j*_ (Additional file 3). Similarly, use case II yielded 32 proteins, 23 of which belonging to *b*_*1*_, 12 to *b*_*1i*_ and 11 to *b*_*1j*_ (Additional file 3). Use case III resulted in six proteins; five of them are members of *b*_*1i*_ (Additional file 3). Finally, use case IV yielded 18 potential regulators of CREB1, three of NFKB1 and two of TCF7L2; all of them are likely DbTFs, based on the TFcheckpoint data (Additional file 3). These regulator proteins were subsequently used in Q6 from use case IV to identify target genes that they share with CREB1, NFKB1 or TCF7L2. This query yielded 20 target genes (19 unique target genes) (Table 2), and were further assessed based on 1) their expression in AR42J cells and 2) their response to gastrin induced stimulation. This finally yielded two target genes that were considered as valid hypotheses (see Table 2 and Figure 1).

**Table 2:** **DbTF** – **target gene categorisation**

**Novel DbTF**	**Function**	**CCK2RDbTF**	**TGs**	**AR42J expressed**	**Gastrin responsive**
**CREM**	Activator	CREB1	JUN	Yes	Yes
**FOXP3**	Repressor	CREB1	IFNG	No	No
	Repressor	CREB1	IL10	No	No
	Repressor	CREB1	BCL2	No	No
	Repressor	CREB1	MALAT1	No	No
**TCF7L2**	Repressor	CREB1	MYOD1	No	No
**FOXP3**	Repressor	NFkB1	PIGR	No	No
	Repressor	NFkB1	CXCL5	No	No
	Repressor	NFkB1	VCAM1	No	No
	Repressor	NFkB1	VWF	No	No
	Repressor	NFkB1	IFNG	No	No
	Repressor	NFkB1	IL8	No	No
	Repressor	NFkB1	BCL2A1	No	No
	Repressor	NFkB1	NFKB1	Yes	Yes
	Repressor	NFkB1	IER3	Yes	Yes
	Repressor	NFkB1	CD40LG	No	No
	Repressor	NFkB1	SELE	No	No
	Repressor	NFkB1	ALOX5AP	Yes	Yes
**SMAD3**	Repressor	NFkB1	MMP9	No	No
**PARP1**	Activator	NFkB1	BRCA2	Yes	Yes

The table lists shared target genes of the novel DbTFs and CCK2R core DbTFs, retrieved through use case I-III. Key for columns (left to right): **Novel DbTFs**: Proteins that transcriptionally regulate the core CCK2R-DbTFs (CREB1, NFkB1 and TCF7L2); **Function**: Role of the regulators; **CCK2R**-**DbTF**: core CCK2R-DbTF that is regulated by the Novel DbTF indicated in column one; **TGs**: Target genes retrieved from GeXKB that are found to be common between the novel DbTFs and the CCK2R core DbTF(s); **AR42J expressed**: known status of target genes expression in AR42J cells [19]; **Gastrin responsive**: known responsiveness of target genes to gastrin treatment [19].

## Discussion

Network based analysis of biological data forms one of the cornerstones of systems biology. Finding new candidate network components is an area of active research [61-63]. Our objective was to demonstrate the use of semantic knowledge bases for such network expansion work, in order to illustrate the potential value of the Semantic Web for biologists. Starting from a literature-based gastrin signaling network [21] that we built previously, we chose three of its documented DNA binding transcription factors (CREB1, NFKB1 and TCF7L2) for the design of a set of biological questions that were formulated as SPARQL queries. This allowed us to retrieve 148 candidate regulators (including the three DbTFs from the query), and 20 shared target genes that are likely to be regulated by both the candidate regulators and the three query DbTFs.

Use case I was designed to identify new activators of CREB1. The only known activator of CREB1 reported in the context of gastrin mediated response is Ribosomal S6 Kinase 1/2 (RSK1/2, see Figure 1), a member of the 90 kDa ribosomal S6 kinase (RSK) protein family [64]. The results obtained from GeXKB suggest several other members of the RSK family to be involved in the activation of CREB1: Ribosomal protein S6 kinase alpha-4 (RPS6KA4) and Ribosomal protein S6 kinase alpha-5 (RPS6KA5), as indicated in Additional file 3 and Figure 1. Our literature search revealed that activation of CREB1 was indeed shown to be regulated by RPS6KA4 and RPS6KA5 [65,66]. However, only RPS6KA4 is expressed in AR42J cells and therefore an interesting candidate for experimental investigation in our gastrin response model system. Similarly, the network candidates PRKD1 (PKD1) and PRKD2 (PKD2) were reported to play a role in CREB1 activation in other cellular responses [67,68], making them interesting candidates for AR42J experiments since they are expressed in this cell line (Additional file 3). Furthermore, repressor candidates TCF7L2, SIRT1 and SIK1 (Additional file 3, and Figure 1) are well documented negative regulators of the CREB1 transcriptional complex in other experimental systems [69-71]. Proteins such as CREB-binding protein (CREBBP, also termed CBP) which have multiple functions depending on the context and environments [72,73], also appear in the query result (see Additional file 3, and Figure 1). This reflects the complexity of the response with various factors interplaying and contributing to CREB1 regulation. Taken together, our analysis of GeXKB for information relevant to the CCK2R network showed that gastrin mediated regulation of CREB1 activity involves several other proteins in addition to RSK1/2, which is the only CREB1-modulator reported so far in the literature. Rather, the cellular outcomes mediated by CREB1 are likely to be dependent on the interplay between different activators such as RPS6KA4 and PRKD1/2 and repressors such as TCF7L2, SIRT1 and SIK1, resulting in fine tuning of CREB1-mediated gene regulatory events triggered by gastrin.

For use case II, literature screening showed that several proteins, including NFΚBIA, CYLD, TAX1BP1, ITCH, SIRT1 and IRAK, have been reported to undergo proteasomal degradation and are implicated in contributing to NFκB down-regulation (see Additional file 3 and references therein, and Figure 1). However, in the gastrin response signaling cascade only NFΚBIA has so far been experimentally shown to be associated with negative regulation of NFκB (reference in Additional file 3, Figure 1). The GeXKB query result suggests additional proteins e.g. CYLD, TAX1BP1, ITCH, SIRT1 and IRAK, that are documented as NFκB repressors undergoing proteasomal degradation (see Additional file 3, and Figure 1) and which can therefore be interesting to pursue in future experimental work.

Interestingly, in use case III the genes encoding these six proteins (PARP1, RUNX3, CTNB1, XRCC5, XRCC6 and DAXX) are all expressed in AR42J cells. Five of these proteins (see Additional file 3 and Figure 1) have literature evidence indicating that they function both as activators and repressors, depending on the context. Of these six proteins, only β-catenin (CTNNB1) has previously been shown to modulate TCF7L2 in gastrin mediated intracellular signaling.

In use cases I-III, protein candidates that show evidence for gastrin induced regulation in the AR42J cell line model system and other model systems (i.e. *b*_*1i*_) were considered as high priority mainly due to the available literature evidence. However, we believe that further investigation of proteins classified under the *b*_*1j*_ category will certainly enhance the identification of novel candidates important for regulating gastrin activated DbTFs.

The result of use case IV based on the TFcheckpoint graph suggests that regulators CREM, FOXP3, TCF7L2, SMAD3 and PARP1 are DbTFs and share 20 target genes that are also regulated by the well-known DbTFs CREB1 and NFkB1 (see Table 2). The genes encoding regulators CREM, TCF7L2 and PARP1 are found to be expressed in AR42J cells. Therefore, potential targets of any of the AR42J expressed regulators would be of greater significance. Further, to identify the potential target genes for experimental validation in response to gastrin, we selected target genes that show change in gene expression during the 14 h gastrin treatment time course in AR42J cells. With this criterion, GeXKB provided the five candidate target genes: JUN, NFkB1, IER3, ALOX5AP and BRCA2 (see Table 2). However, among these genes, only JUN and BRCA2 are identified as being targets of both regulators (CREM and PARP1, Figure 1).

The results presented in this paper demonstrate that GeXKB can facilitate the identification of potential novel regulators of gastrin activated DbTFs. Based on our results with gastrin-mediated gene regulation reported in the present paper, we believe that GeXKB can be of equal use in any other experimental system as well. Obviously, the more specific biological roles of the regulators and target genes identified through GeXKB require further experimental validation. Observations made through small scale experiments such as RNAi mediated knock down of the novel regulators or large-scale studies on knock out model organisms should greatly enhance our current understanding of transcription regulation and subsequent cellular outcomes. Information contained in gene expression databases such as ArrayExpress may provide clues as to the role of genes and products thereof. We therefore searched for gene knockout experiments concerning the gastrin regulation network candidates in ArrayExpress and found evidence for candidate regulators CRTC1 and COMD1 (see Figure 1, where these are represented by *yellow* and *turquoise* nodes respectively): gene knock-out experiments conducted on CRTC1 and COMD1 implicate them as a potential regulator of transcription of CREB1 (ArrayExpress accession: E-GEOD-12209) and NFkB1 (ArrayExpress accession: E-MEXP-832), respectively.

## Conclusions

Our work demonstrates the level of knowledge discovery that can be achieved when information from a broad range of GO annotations and experimental evidence is semantically integrated. Interlinking various data sets using RDF provides the much needed homogeneity and extensibility for advanced data analysis. Additionally, we have shown the benefits of using computational inferencing in building the knowledge base, as this approach allows the retrieval of information that would otherwise have remained implicit and hidden from querying. Our efforts have involved a close collaboration between Semantic Web specialists and biological domain experts, resulting in novel ways for generating hypotheses and an initial assessment of these hypotheses against the current understanding of a regulatory network.

The utility of GeXKB is expected to grow with its further development. The goal for future releases will be to expand the knowledge base with additional high quality datasets which will include relations between DbTFs and other interactors from curated texts, partially based on our current work on checking the full repertoire of transcription factors of human, mouse and rat, and their respective target genes.

## Additional files

Additional file 1
**GeXKB metrics.** The sheets; ‘Terms’ and ‘Relations’ provide summaries of the three application ontologies; Spreadsheet ‘TFs-TGs’ provides metrics for the additional sources. Additional file 2
**SPARQL_queries.** This file lists the 6 SPARQL queries (Q1- Q6) formulated for use cases I – IV. Additional file 3
**Query_results.** This spreadsheet lists the results returned for queries Q1 – Q5. The proteins are annotated according to the query, evaluation categories and evidence.

## Abbreviations

GeXKB: Gene eXpression Knowledge Base
GeXO: Gene eXpression Ontology
ReXO: Regulation of Gene eXpression Ontology
ReTO: Regulation of Transcription Ontology
ULO: Upper Level Ontology
OBO: Open Biomedical Ontologies
GO: Gene Ontology
RDF: Resource Description Framework
RDFS: RDF Schema
OWL: Web Ontology Language
SPARQL: SPARQL query language
SPARUL: SPARQL update language
URI: Uniform Resource Identifiers
HTTP: Hypertext Transfer Protocol
CCO: Cell Cycle Ontology
OBI: Ontology for Biomedical Investigations
ChEBI: Chemical Entities of Biological Interest
IAO: Information Artifact Ontology
PSI-MOD: Proteomics Standards Initiative-Protein Modification
RDBMS: Relational Database Management System
PDB: Protein Data Bank
HTRI: Human Transcriptional Regulation Interactions
DbTF: DNA binding transcription factor
CCK2R: Cholecystokinin 2 receptor
CREB1: cAMP response element binding protein 1
CREBBP: CREB-binding protein
RSK: Ribosomal S6 Kinase
CTNNB: Beta-catenin
IkB: Inhibitors of kB
