# Supplementary material for: Finding gene regulatory network candidates using the gene expression knowledge base
Source: BMC Bioinformatics. 2014 Dec 10;15(1):386. doi: 10.1186/s12859-014-0386-y (PMC4279962; doi:10.1186/s12859-014-0386-y)
Supplement: Additional file 1 — GeXKB metrics. The sheets; ‘Terms’ and ‘Relations’ provide summaries of the three application ontologies; Spreadsheet ‘TFs-TGs’ provides metrics for the additional sources. [file 12859_2014_386_MOESM1_ESM.docx]

## GeXKB metrics:

Table ***a*** and ***b*** list the relations (predicates) and numbers of entries associated with different term types found in the three application ontologies. Table ***c*** lists the number of RDF triples, DbTFs and target genes (TGs) from secondary resource graphs.

|  | **GO** | **MI** | **intact** | **KEGG** | **UniProtKB** | **SSB** | **NCBIGene** | **NCBITaxon** | **ULO** |
| --- | --- | --- | --- | --- | --- | --- | --- | --- | --- |
| **GeXO** | 12936 | 69 | 17714 | 2278 | 37237 | 108 | 19369 | 3 | 20 |
| **ReXO** | 12549 | 69 | 14918 | 1769 | 31187 | 106 | 16988 | 3 | 20 |
| **ReTO** | 12313 | 69 | 12903 | 1506 | 27978 | 99 | 15330 | 3 | 20 |

***a)***

***b)***

| **Subject namespace** | **Predicate** | **Object namespace** | **GeXO** | **ReXO** | **ReTO** | **Comments** |
| --- | --- | --- | --- | --- | --- | --- |
| NCBIGene | codes_for | UniProtKB | 24209 | 21106 | 18872 |  |
| GO | contains | UniProtKB | 82396 | 71309 | 63748 | Cellular Component |
| intact | has_agent | UniProtKB | 49076 | 42326 | 37066 |  |
| UniProtKB | has_function | GO | 110214 | 98309 | 89542 | Molecular Function |
| GO | has_participant | UniProtKB | 37957 | 23033 | 19833 | Biological Process |
| UniProtKB/ NCBIGene | has_source | NCBITaxon | 51437 | 43296 | 39009 |  |
| UniProtKB | member_of | KEGG | 15061 | 12813 | 11136 |  |
| SSB | occurs_in | UniProtKB | 17462 | 15616 | 13924 |  |
| UniProtKB | orthologous_to | UniProtKB | 46182 | 36870 | 32332 |  |
| UniProtKB | paralogous_to | UniProtKB | 21862 | 18040 | 16498 |  |

***c)***

|  | **Triples** | **DbTFs** | **TGs** |
| --- | --- | --- | --- |
| **HTRIdb** | 717440 | 468 | 18302 |
| **TFactS** | 98142 | 1016 | 3376 |
| **PAZAR** | 107281 | 453 | 4419 |
| **TFcheckpoint** | 151283 | 3727 | n/a |
