# Supplementary material for: Finding gene regulatory network candidates using the gene expression knowledge base
Source: BMC Bioinformatics. 2014 Dec 10;15(1):386. doi: 10.1186/s12859-014-0386-y (PMC4279962; doi:10.1186/s12859-014-0386-y)
Supplement: Additional file 2 — SPARQL_queries. This file lists the 6 SPARQL queries (Q1- Q6) formulated for use cases I – IV. [file 12859_2014_386_MOESM2_ESM.pdf]

Supplementary material

Q1:

Biological Question: List of proteins involved in activation of CREB1 Transcription factor

Parameters:

- GO_0032793 - positive regulation of CREB transcription factor activity,
- GO_0051091 - positive regulation of sequence-specific DNA binding transcription factor activity + MI_0914 – association,
- MI_0407 - Direct interactors,
- GO_0008140 - cAMP response element binding protein binding

SPARQL query:

```
BASE   <http://www.semantic-systems-biology.org/>
PREFIX rdfs:<http://www.w3.org/2000/01/rdf-schema#>
PREFIX ssb:<http://www.semantic-systems-biology.org/SSB#>

PREFIX term: <SSB#UniProtKB_P16220> # CREB1

SELECT distinct ?gene ?name ?description ?dbtf ?protein
WHERE {
 GRAPH <ReTO> {
  ?protein ssb:has_source ssb:NCBITaxon_9606 .
  ?protein ssb:Definition ?d .
  ?d ssb:def ?description .
  ?g ssb:codes_for ?protein .
  ?g rdfs:label ?gene .
  ?protein rdfs:label ?name .
 }
 {
  GRAPH <ReTO> {
   ssb:GO_0032793 ssb:has_participant ?protein .
  }
 }UNION {
  GRAPH <ReTO-tc> {
   ?biological_process ssb:is_a ssb:GO_0051091 .
   ?biological_process ssb:has_participant ?protein .
   ?interaction ssb:is_a ssb:MI_0914 .
   ?interaction ssb:has_agent ?protein .
   ?interaction ssb:has_agent term: .
  }
 } UNION {
  GRAPH <ReTO-tc> {
   ?interaction ssb:is_a ssb:MI_0407 .
   ?interaction ssb:has_agent ?protein .
   ?interaction ssb:has_agent term: .
  }
 } UNION {
  GRAPH <ReTO> {
   ?protein ssb:has_function ssb:GO_0008140 .
  }
 }
   FILTER (?protein != term:)
 OPTIONAL {
  GRAPH <tfcheckpoint> {
   ?protein ssb:is_dbtf ?dbtf.
  }
 }
}

ORDER BY ?gene
```

Q2:

Biological Question: Name transcriptional repressors of CREB1 Transcription factor

Parameters:

- GO_0043433 - negative regulation of sequence-specific DNA binding transcription factor activity,
- GO_0032792 - negative regulation of CREB transcription factor activity

SPARQL query:

```
BASE   <http://www.semantic-systems-biology.org/>
PREFIX rdfs:<http://www.w3.org/2000/01/rdf-schema#>
PREFIX ssb:<http://www.semantic-systems-biology.org/SSB#>

SELECT distinct ?gene ?name ?description ?dbtf ?repressor
WHERE {
  GRAPH <ReTO> {
   ?repressor ssb:has_source ssb:NCBITaxon_9606 .
   ?repressor rdfs:label ?name .
   ?repressor ssb:Definition ?d .
   ?d ssb:def ?description .
   ?g ssb:codes_for ?repressor .
   ?g rdfs:label ?gene .
  }
  GRAPH <ReTO> {
   {
    ssb:GO_0043433 ssb:has_participant ?repressor .
   } UNION {
    ssb:GO_0032792 ssb:has_participant ?repressor .
   }
  }
  OPTIONAL {
  GRAPH <tfcheckpoint> {
   ?repressor ssb:is_dbtf ?dbtf.
  }
 }
}

ORDER BY ?gene
```

Q3:

Biological Question: List chromatin modifiers which are part of CREB transcription factor complex

Parameters:

- GO_0004402 - histone acetyltransferase activity,
- GO_0004407 - histone deacetylase activity,
- GO_0051090 - regulation of sequence-specific DNA binding transcription factor activity
- GO_0005667 - transcription factor complex + MI_0914 - association

SPARQL Query:

```
BASE   <http://www.semantic-systems-biology.org/>
PREFIX rdfs:<http://www.w3.org/2000/01/rdf-schema#>
PREFIX ssb:<http://www.semantic-systems-biology.org/SSB#>

PREFIX term: <SSB#UniProtKB_P16220> # CREB1-P16220

SELECT distinct ?gene ?chromatin_modifier_name ?description ?chromatin_modifier
WHERE {
 GRAPH <ReTO> {
  ?chromatin_modifier ssb:has_source ssb:NCBITaxon_9606 .
```

```
  ?chromatin_modifier ssb:Definition ?Def .
  ?Def  ssb:def   ?description .
  ?chromatin_modifier rdfs:label ?chromatin_modifier_name .
  ?g ssb:codes_for ?chromatin_modifier .
  ?g rdfs:label ?gene .
 }
 GRAPH <ReTO-tc> {
  {
    ?chromatin_modifier ssb:has_function ?function .
    ?function ssb:is_a ssb:GO_0004402 .
  } UNION {
    ?chromatin_modifier ssb:has_function ?function .
    ?function ssb:is_a ssb:GO_0004407 .
  }
  ssb:GO_0051090 ssb:has_participant ?chromatin_modifier .
 }
 GRAPH <ReTO-tc> {
  ?complex ssb:is_a ssb:GO_0005667 .
  ?complex ssb:contains ?chromatin_modifier .
  ?complex ssb:contains term: .
  OPTIONAL {
   ?interaction ssb:is_a ssb:MI_0914 .
   ?interaction ssb:has_agent term: .
   ?interaction ssb:has_agent ?chromatin_modifier .
  }
 }
}

ORDER BY ?gene
```

Q4:

Biological Question: List all transcriptional repressors of Transcription factors NFkB1 and RELA which undergoes proteosomal degradation.

Parameters:

- GO_0032088 - negative regulation of NF-kappaB transcription factor activity
- KEGG_ko04120 - Ubiquitin mediated proteolysis,
- GO_0000151 - ubiquitin ligase complex,
- GO_0043130 - ubiquitin binding,
- MI_0220 - ubiquitination reaction

SPARQL Query:

```
BASE   <http://www.semantic-systems-biology.org/>
PREFIX rdfs:<http://www.w3.org/2000/01/rdf-schema#>
PREFIX ssb:<http://www.semantic-systems-biology.org/SSB#>

SELECT distinct ?gene ?name ?description ?dbtf ?protein
WHERE {
  GRAPH <ReTO> {
  ?protein ssb:has_source ssb:NCBITaxon_9606 .
  ?protein rdfs:label ?name .
  ?protein ssb:Definition ?d .
  ?d ssb:def ?description .
  ?g ssb:codes_for ?protein .
  ?g rdfs:label ?gene .
 }
 {
  GRAPH <ReTO-tc> {
   ?cellular_component ssb:is_a ssb:GO_0000151 .
   ?cellular_component ssb:contains ?protein .
  }
  GRAPH <ReTO> {
```

```
   ssb:GO_0032088 ssb:has_participant ?protein .
  }
 }
 UNION {
  GRAPH <ReTO> {
   ssb:GO_0032088 ssb:has_participant ?protein .
  }
  GRAPH <ReTO> {
   ssb:KEGG_ko04120 ssb:has_agent ?protein_cluster .
   ?q_prot ssb:is_member_of ?protein_cluster .
  }
  GRAPH <ReTO> {
   ?interaction ssb:is_a ssb:MI_0915 .
   ?interaction ssb:has_agent ?protein .
   ?interaction ssb:has_agent ?q_prot .
  }
 }
 UNION {
  GRAPH <ReTO-tc> {
   ?function ssb:is_a ssb:GO_0043130 .
   ?protein ssb:has_function ?function .
   ssb:GO_0032088 ssb:has_participant ?protein .
  }
 }
 UNION {
  GRAPH <ReTO-tc> {
   ?interaction ssb:is_a ssb:MI_0220 .
   ?interaction ssb:has_agent ?protein .
   ssb:GO_0032088 ssb:has_participant ?protein .
  }
 }
  OPTIONAL {
  GRAPH <tfcheckpoint> {
   ?protein ssb:is_dbtf ?dbtf.
  }
 }
}

ORDER BY ?gene
```

Q5:

Biological Question: List all transcriptional repressors of TCF7L2 which are activators of NFkB1 or CREB1.

Parameters:

- GO_0043433 - negative regulation of sequence-specific DNA binding transcription factor activit
- GO_0051091 - positive regulation of sequence-specific DNA binding transcription factor activity,
- MI_0914 - association

SPARQL Query:

```
BASE <http://www.semantic-systems-biology.org/>
PREFIX rdfs:<http://www.w3.org/2000/01/rdf-schema#>
PREFIX ssb:<http://www.semantic-systems-biology.org/SSB#>

PREFIX term: <SSB#UniProtKB_Q9NQB0> # TCF7L2

SELECT distinct ?gene ?name ?description ?dbtf ?protein
WHERE {
 GRAPH <ReTO> {
  ?protein ssb:has_source ssb:NCBITaxon_9606 .
  ?protein rdfs:label ?name .
  ?protein ssb:Definition ?d .
```

```
  ?d ssb:def ?description .
  ?g ssb:codes_for ?protein .
  ?g rdfs:label ?gene .
 }
 GRAPH <ReTO-tc> {
  ?interaction ssb:is_a ssb:MI_0914 .
  ?interaction ssb:has_agent term: .
  ?interaction ssb:has_agent ?protein .
  FILTER (?protein != term:)
 }
 GRAPH <ReTO-tc> {
  ?biological_process1 ssb:is_a ssb:GO_0043433 .
  ?biological_process1 ssb:has_participant ?protein .
  ?biological_process2 ssb:is_a ssb:GO_0051091 .
  ?biological_process2 ssb:has_participant ?protein .
 }
  OPTIONAL {
  GRAPH <tfcheckpoint> {
   ?protein ssb:is_dbtf ?dbtf.
  }
 }
}

ORDER BY ?gene
```

Q6:

Biological Question: Identification of shared target genes between regulators and their DbTFs

Parameters:
- Regulators retrieved from Q1, Q2, Q4 and Q5 that are DbTF,
- DbTF of interest (CREB1, NFKB1 and TCF7L2)

SPARQL Query:

```
BASE   <http://www.semantic-systems-biology.org/>
PREFIX rdf:<http://www.w3.org/1999/02/22-rdf-syntax-ns#>
PREFIX rdfs:<http://www.w3.org/2000/01/rdf-schema#>
PREFIX ssb:<http://www.semantic-systems-biology.org/SSB#>

PREFIX reg_term:<SSB#UniProtKB_Q9BZS1>  # FOXP3, DbTF terms to be changed
accordingly

PREFIX creb:<SSB#UniProtKB_P16220> # CREB1 protein term
PREFIX nfkb:<SSB#UniProtKB_P19838> # NFKB1 protein term
PREFIX tcf7l2:<SSB#UniProtKB_Q9NQB0> # TCF7L2 protein term

SELECT distinct ?name ?tg
WHERE {
 {
  GRAPH <htridb> {
   reg_term: ssb:acts_on ?tg.
   creb: ssb:acts_on ?tg.
#   nfkb: ssb:acts_on ?tg.
#   tcf7l2: ssb:acts_on ?tg.
   ?tg rdfs:label ?name.
  }
 }
 UNION {
  GRAPH <tfacts> {
   reg_term: ssb:acts_on ?tg.
   creb: ssb:acts_on ?tg.
```

```
#   nfkb: ssb:acts_on ?tg.
#   tcf7l2: ssb:acts_on ?tg.
   ?tg rdfs:label ?name.
  }
 }
 UNION {
  GRAPH <UP-IDMAP> {
   reg_term: ssb:ensembl_trs ?term_mrna.
   creb: ssb:ensembl_trs ?creb_mrna.
#   nfkb: ssb:ensembl_trs ?nfkb_mrna.
#   tcf7l2: ssb:ensembl_trs ?tcf7l2_mrna.
  }
  GRAPH <PAZAR> {
   ?term_mrna ssb:acts_on ?tg.
   ?creb_mrna ssb:acts_on ?tg.
#   ?nfkb_mrna ssb:acts_on ?tg.
#   ?tcf7l2_mrna ssb:acts_on ?tg.
   OPTIONAL {
     GRAPH ?g {
      ?tg rdfs:label ?name.
     }
   }
  }
 }
}
```
